# SAMHD1 as a prognostic and predictive biomarker in stage II colorectal cancer: A multicenter cohort study

**DOI:** 10.3389/fonc.2022.939982

**Published:** 2022-08-01

**Authors:** Dingyun You, Shuai Zhang, Shan Yan, Yingying Ding, Chunxia Li, Xianshuo Cheng, Lin Wu, Weizhou Wang, Tao Zhang, Zhenhui Li, Yongwen He

**Affiliations:** ^1^ Department of Dental Research, The Affiliated Stomatological Hospital of Kunming Medical University, Kunming, China; ^2^ Yunnan Key Laboratory of Stomatology, Kunming Medical University, Kunming, China; ^3^ Yunnan Key Laboratory of Stem Cell and Regenerative Medicine, Biomedical Engineering Research Center, Kunming Medical University, Kunming, China; ^4^ Department of Biostatistics, School of Public Health, Cheeloo College of Medicine, Shandong University, Jinan, China; ^5^ Department of Radiology, The Third Affiliated Hospital of Kunming Medical University, Yunnan Cancer Hospital, Yunnan Cancer Center, Kunming, China; ^6^ Department of Colorectal Surgery, The Third Affiliated Hospital of Kunming Medical University, Yunnan Cancer Hospital, Yunnan Cancer Center, Kunming, China; ^7^ Department of Pathology, The Third Affiliated Hospital of Kunming Medical University, Yunnan Cancer Hospital, Yunnan Cancer Center, Kunming, China; ^8^ Department of Orthopedics, The First Affiliated Hospital of Kunming Medical University, Kunming, China

**Keywords:** SAMHD1, colorectal cancer, Cox model, prognostic markers, nested case-control design, MSI

## Abstract

**Background:**

The identification of high-risk population patients is key to the personalized treatment options for the stage II colorectal cancers. The use of proteomics in the prognosis of patients with stage II colorectal cancer remains unclear.

**Methods:**

Using quantitative proteomics, we analyzed proteins that are differentially expressed in the tumor and adjacent normal tissues of 11 paired colorectal cancer patients with and without recurrence selected by a nested case-control design. Of the 21 identified proteins, we selected one candidate protein. The association of the corresponding gene of the selected protein with overall survival (OS) and adjuvant chemotherapy was analyzed using two independent cohorts of patients with stages II colorectal cancer.

**Results:**

Sterile α motif and histidine-aspartate domain-containing protein 1 (SAMHD1) was selected as the candidate biomarker. A group of 124 patients (12.5%) were stratified into SAMHD1-high subgroup. The 5-year OS rate of SAMHD1-high patients was lower than that of SAMHD1-low patients with stage II colorectal cancer (discovery cohort: hazard ratio [HR] = 2.89, 95% confidence interval [CI], 1.17-7.18, *P* = 0.016; validation cohort: HR = 2.25, 95% CI, 1.17-4.34, *P* = 0.013). The Cox multivariate analysis yielded similar results. In a pooled database, the 5-year OS rate was significantly different between patients with and without adjuvant chemotherapy among stage II SAMHD1-low tumors than in patients with stage II SAMHD1-high tumors (88% vs. 77%, *P* = 0.032).

**Conclusions:**

SAMHD1-high expression could help in identifying patients with stage II colorectal cancer with poor prognosis and less benefit from adjuvant chemotherapy.

## Introduction

Globally, colorectal cancer is the third most common malignant tumor and the second leading cause of cancer-related deaths (1). In China, colorectal cancer poses a huge health burden, with more than 290,000 deaths reported annually (2). Local recurrence and distant metastasis are the major reasons for the high mortality rate in patients with resectable colorectal cancer (3). The rate of recurrence for stages II and III colorectal cancer is approximately 20% and 48%, respectively (4). To reduce the incidence of recurrence, adjuvant chemotherapy following total meso-rectal excision is the standard of care for stage III patients according to international guidelines (5–8); wherein patients with stage III colorectal cancer who received adjuvant chemotherapy showed significant improvement in survival (9). However, patients with stage II colorectal cancer showed minimal improvement in the 5-year overall survival (OS) rate (2%–5%) (10). There is no consensus on whether patients with stage II colorectal cancer could benefit from adjuvant chemotherapy; therefore, recommending adjuvant chemotherapy for those patients is still controversial (5–8). Therefore, it is crucial to identify patients with stage II colorectal cancer who could benefit from adjuvant chemotherapy.

Prognostic risk factors are essential to help clinicians make better-informed decisions while selecting the best treatment strategy for patients with stage II colorectal cancer and determining the need for adjuvant treatment (11). At present, the major well-known prognostic risk factors for patients with colorectal cancer are stage pT4, bowel perforation or occlusion, lymphatic-vascular-perineural invasion, poorly differentiated histology (excluding microsatellite instability-high [MSI-H] tumors), inadequate lymph node sampling, and positive margins after surgery (5–8). However, all these factors, except stage pT4, are insufficient to identify patients with stage II colorectal cancer who could benefit from adjuvant chemotherapy (12). In the last few years, several efforts have been made to identify novel biomarkers that are able to predict a higher risk of relapse in patients with stage II colorectal cancer, such as identifying their gene expression signatures (Oncotype, ColoPrint, ColDX) (13–15), microRNA signatures (16), circulating tumor DNA (17–21), immune-related signatures (22–24), and deep learning signatures (25). However, high costs or complexity in the techniques of these approaches have prevented their successful translation into routine clinical practice. This has led to the emerging need for the identification of novel and more feasible biomarkers.

Over the past few years, mass-spectrometry-based proteomics has emerged as the method of choice for identifying possible prognostic indicators of outcome and disease response to therapy (26–29). We used proteomics to identify and select sterile α motif and histidine-aspartate domain-containing protein 1 (SAMHD1) as the candidate biomarker based on literature reviews and experiments. Using subgroup analysis involving retrospective patient cohorts, we evaluated the association between the SAMHD1 biomarker and the benefits from adjuvant chemotherapy and survival in patients with stage II and III colorectal cancer.

## Methods

### Patients and samples

**Figure 1:**
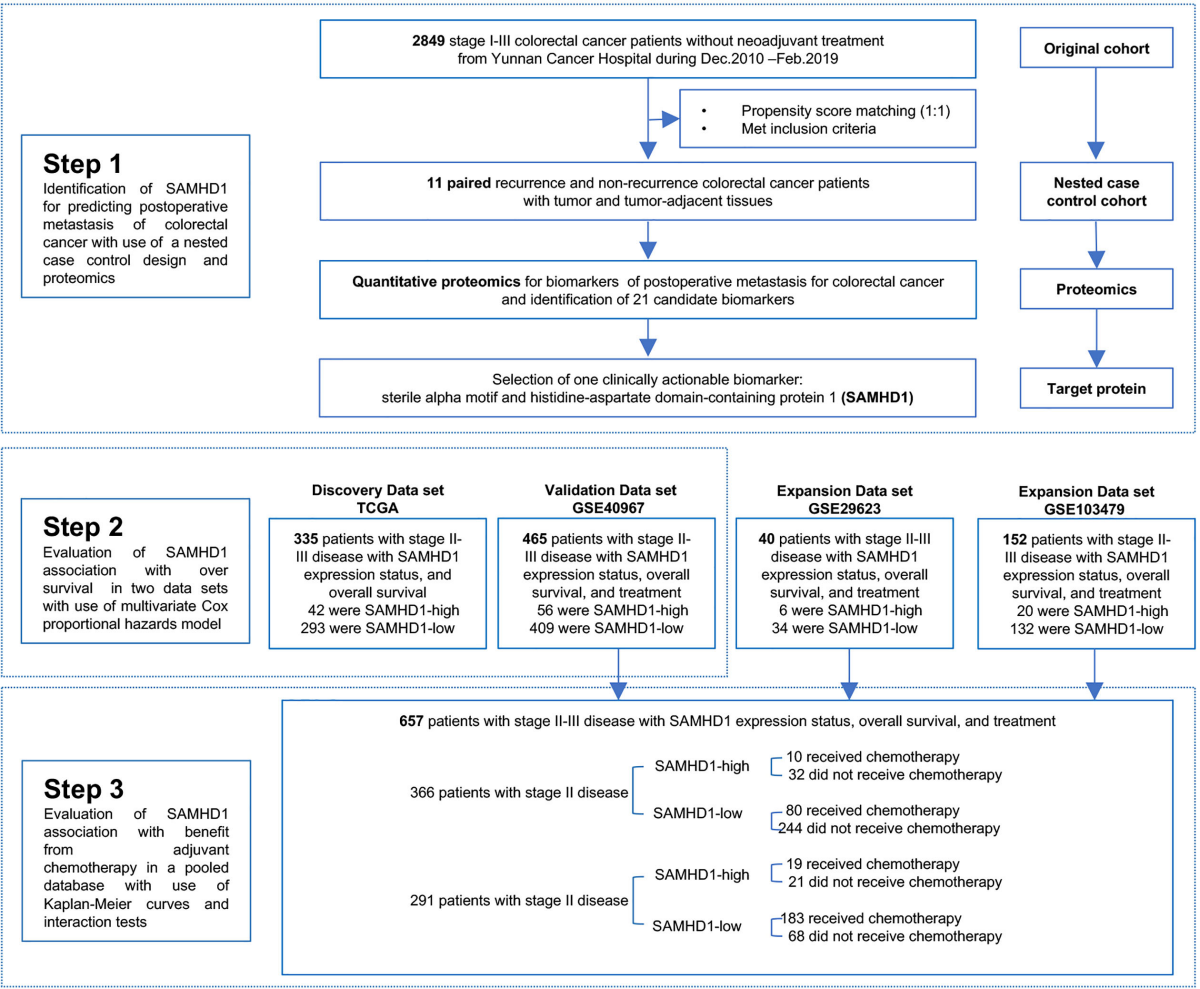
Study Design. PSM, propensity score matching; SAMHD1, sterile α motif and histidine-aspartate domain-containing protein 1; TCGA, The Cancer Genome Atlas; GEO, Gene Expression Omnibus.

The study protocol was approved by the Yunnan Cancer Hospital Ethics Committee (No. KY2019141). The requirement for informed consent was waived by the ethics committee owing to the retrospective nature of the study. The data were anonymized. For proteomic analysis, surgically resected biopsies of patients with colorectal cancer and paired non-cancerous tissues (collected 10 cm from the tumor) were collected from 11 pairs of patients with stage II and III colorectal cancer with and without recurrence, from Yunnan Cancer Hospital. These 11 pairs of patients were selected by propensity score matching (PSM) from the original cohort, including consecutive patients with stage I–III colorectal cancer who underwent radical resection at Yunnan Cancer Hospital between December 2010 and February 2019 (referred to as the Yunnan colorectal cancer cohort). The association between the expression levels of SAMHD1 messenger RNA (mRNA) and OS was tested in a discovery dataset of 335 patients from The Cancer Genome Atlas (TCGA) and a validation dataset of 465 patients from the National Center for Biotechnology Information Gene Expression Omnibus (NCBI-GEO). Patients who received neoadjuvant treatment were excluded from the analysis. The flowchart of the study is shown in **Figure 1**.

### Propensity score matching

We performed PSM (30, 31) to strictly balance the critical variables between postoperative and non-postoperative metastatic patients within 3 years after surgery in the Yunnan colorectal cancer cohort. Propensity scores were generated using a logistic regression model with age, sex, body mass index, surgical pathological type, site of primary carcinoma, and pathological stage as the independent variables. Each metastatic patient was matched 1:1 to two patients in the non-metastasis group using a 0.001 caliper width (propensity scores must be within 0.1% of each other to create a match), and the resulting matches were used in the following selection.

Subsequently, we selected matched patients according to the following inclusion criteria: (1) patients with stage II or III colorectal cancer; (2) available formalin-fixed paraffin-embedded (FFPE) specimens; (3) available data on recurrence-free survival (RFS) and OS; (4) patients without recurrence whose duration of OS is longer than that of patients with recurrence; and (5) data including cancerous and paired non-cancerous tissues. Tandem mass tag (TMT)-labelled quantitative proteomics was performed on the matched patients.

### TMT-labelling quantitative proteomics

For each patient, quantitative proteomics was performed on the tumor and tumor-adjacent tissues, and the protein was extracted using the FFPE Total Protein Extraction Kit (Sangon Biotech, NO. C500058, Shanghai, China), according to the manufacturer’s instructions. The extracted proteins were quantified using a BCA protein assay kit (Bio-Rad, USA). Protein digestion was performed according to the FASP procedure described by Wisniewski et al. (32), and the resulting peptide mixture was labeled using the 6-plex TMT reagent according to the manufacturer’s instructions (Thermo Fisher Scientific, Waltham, USA). Detailed procedures for TMT labeling, peptide fractionation, and LC-MS/MS analysis are described in the **Supplementary material A**.

### Identification of the target protein

The differentially expressed proteins between tumor and tumor-adjacent tissues were identified using the Student’s t-test (*P* < 0.05). Proteins associated with metastasis were verified using the univariate Cox regression analysis (*P* < 0.01). Subsequently, we focused on the intersection of the differentially expressed and metastasis-related proteins. Proteins that had rarely been reported in most cancers, according to the literature search and our basic research, were selected for further analysis.

### Analysis of tissue microarrays in the discovery and validation datasets

Gene expression profiles for colorectal cancer tissues, fully annotated with clinical and pathological information, were obtained from two independent sources; TCGA (**Figure S1**) and NCBI-GEO, including GSE40967 (**Figure S2**). A detailed description of the patient cohorts represented by the two independent sources is provided in **Table S1**.

Due to the considerable variation in the coverage of the sequencing platforms, pipelines, assays, and tools/algorithms between the TCGA and GEO datasets, the frequency of the identified variants was impacted (33). Taking these constraints into consideration, we used the Z-score (34) to standardize data across different experiments and to normalize the expression data of SAMHD1 from these two datasets prior to data analysis.

Subsequently, SAMHD1 expression levels were stratified into SAMHD1-high and SAMHD1-low subgroups according to the SAMHD1 expression, the threshold of which was identified in patients with stage II colorectal cancer using X-tile from the discovery dataset (35). We explored the association between the expression levels of SAMHD1, the OS outcomes, and the interaction between SAMHD1 expression level and adjuvant chemotherapy in stage II and stage III colorectal cancer.

### SAMHD1 expression and benefit from adjuvant chemotherapy

To evaluate whether patients with SAMHD1-high tumors could benefit from adjuvant chemotherapy, we investigated the association between SAMHD1 status (assessed at the mRNA level) and OS among patients who either did or did not receive adjuvant chemotherapy in the NCBI-GEO dataset by pooling the following three datasets: GSE40967, GSE29623 and GSE103479. The three datasets were found to satisfy our criteria (i.e., knowledge of pathological stage, available information on SAMHD1 expression, adjuvant chemotherapy, duration of OS, and follow-up duration) (**Figures S3**, **S4**, and **Table S1**).

### Statistical analysis

We downloaded the transcriptome profiles in FPKM format and the corresponding clinical information from the TCGA portal (https://portal.gdc.cancer.gov/) and NCBI-GEO dataset (https://www.ncbi.nlm.nih.gov/geo/). The NCBI-GEO datasets recruited for multiple dataset analysis and were based on different platforms. Therefore, we combined the three datasets to expand the sample size and avoid generating less reliable results by normalization using the robust multi-chip average (RMA) algorithm and removed the batch effect using the affy and sva R packages. Probes corresponding to the same gene were averaged.

Patient subgroups were compared with respect to survival outcomes using Kaplan–Meier curves, log-rank tests, and multivariate analyses based on the Cox proportional hazards method adjusting for patient age, sex, and adjuvant chemotherapy.

All analyses were conducted using R software (version 3·6·3; http://www.R-project.org). Statistical significance was set at *P* < 0.05.

## Results

### The clinical characteristics of 11 paired-patients

Eleven pairs of patients were selected through PSM. The clinical and pathological characteristics of patients with stage II or III colorectal cancer are shown in **Table S2**. The age of the patients in the non-metastasis group ranged between 46 and 74 years. In the non-metastatic group, 5 patients were males, 8 patients were in stage II, one patient died, and no recurrence occurred. The median OS and RFS follow-up times were both 51.6 months. The age of patients in the metastatic group ranged between 42 and 75 years. In the metastatic group, five patients were males, eight patients had stage II disease, five patients died, and recurrence occurred in five patients. The median OS and RFS follow-up times were 31.8 and 21.4 months, respectively. Based on the univariate analysis, there was a significant difference in the OS follow-up time (51.6 months vs 31.8 months, respectively, *P* = 0.002), RSF event (100% vs 54.5%, respectively, *P* = 0.042), and RFS follow-up time (51.6 months vs 21.4 months, respectively, *P* < 0.001) between the non-metastatic and metastatic groups. **Figure S5** shows the OS and RFS curves for all patients.

### Identification of SAMHD1

A total of 5,197 proteins were identified using TMT-labelled quantitative proteomics. We processed the protein expression data by deleting proteins in which more than 50% of the samples had missing values. Among the remaining 2,760 proteins, which were retained for further analysis, 1,409 proteins showed significance with the *P* < 0.05 t-test threshold (**Table S3**); 38 proteins were associated with metastasis (*P* < 0.01) in the univariate Cox regression analysis (**Table S4**). The volcano plot shows the distribution of *P*-values of the t-test and the univariate Cox regression analysis (**Figure S6A**). In our study, 28 candidate proteins were found to be common between the 1,409 differentially expressed proteins and the 38 metastasis-related proteins (**Figure S6B**). Of the 28 candidate proteins, 7 proteins were not annotated with coding genes. The information regarding the remaining 21 proteins is shown in **Table S5**.

Based on previous literature reviews and basic experiments, we screened these 21 proteins and finally yielded the protein SAMHD1 (36–39). **Figure S7** shows the different distributions of SAMHD1 expression in the cancer tissues between the non-metastatic and metastatic groups.

The Pearson’s correlation coefficients between the expression of the SAMHD1 gene and the genes associated with microsatellite instability (MLH1, MSH2, MSH6, and PMS2) were -0.012, 0.17, 0.19, and 0.28, the results indicated there was a weak correlation between SAMHD1 expression and microsatellite instability-related genes (**Figure S8**), and SAMHD1 has a good complementary effect with those genes. Additionally, we compared and contrasted SAMHD1 expression according to KRAS mutation status, BRAF mutation status, tumor location, and defective DNA mismatch repair status, as they were frequently mutated genes or risk parameters in colorectal cancer. Statistical significance was detected using t-test for comparisons between all those groups. The results showed that SAMHD1 gene expression only partially overlapped with tumors defined by those factors (**Figure S9**).

### SAMHD1 expression and OS in the discovery dataset

The optimum cutoff score for SAMHD1 expression generated by the X-tile plot was 1.15 (**Figure S10**). In total, the 335 patients were stratified into SAMHD1-low (n = 293, 87.5%) and SAMHD1-high (n = 42, 12.5%) groups, according to the expression of SAMHD1 in the discovery dataset. The baseline characteristics and known molecules of stage II and III in the discovery dataset were shown in **Table S6**.

**Figure 2:**
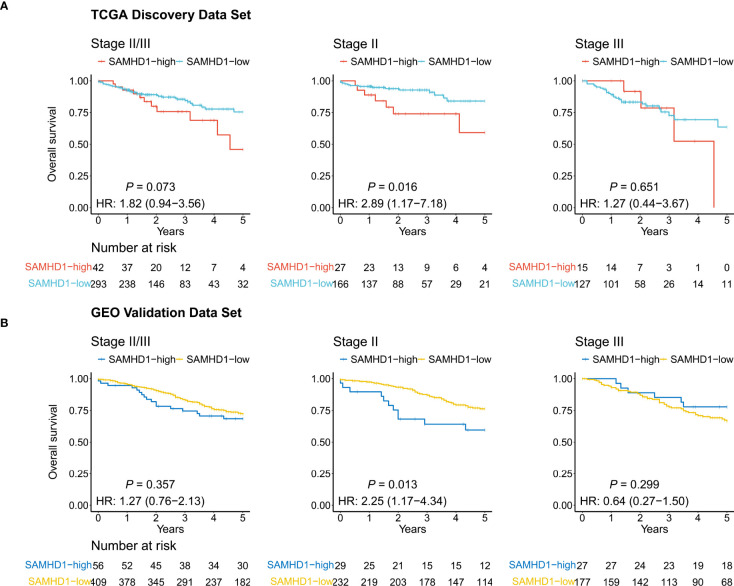
Kaplan–Meier curves. The curves show the relevance between the 5-year overall survival and SAMHD1 gene expression status in colorectal cancer, using The Cancer Genome Atlas data (TCGA) **(A)** and Gene Expression Omnibus data (GEO) **(B)**. Left: Patients with stage II and III disease. Middle: Patients with stage II disease. Right: Patients with stage III disease.

Kaplan–Meier curves were used to compare the 5-year OS of the two groups. As shown in **Figure 2A**, the expression level of SAMHD1 tended to be associated with the 5-year OS (SAMHD1-high vs. SAMHD1-low; HR = 1.82; 95% CI, [0.94–3.56]; and *P* = 0.073) among patients with stage II and III colorectal cancer. With respect to stage II, the 5-year OS rate of the 27 patients (13.99%) with SAMHD1-high expression level was higher than that that of the 166 patients (86.01%) with SAMHD1-low expression level (HR = 2.89; 95% CI, [1.17–7.18]; and *P* = 0.016). However, there was no significant difference in the 5-year OS between the SAMHD1-low (n = 127) and SAMHD1-high (n = 15) groups with stage III colorectal cancer (HR = 1.27; 95% CI, [0.44–3.67]; and *P* = 0.651). In the multivariate analysis, with adjustment of age and sex as confounding variables, the HR for OS among stage II patients with SAMHD1-high versus SAMHD1-low was 2.99 (95% CI, [1.17–7.65]; and *P* = 0.023) (**Table 1**).

**Table 1:** Univariate and multivariable Cox analyses for overall survival among patients in the discovery data set.

Subgroup	Variable	Univariate Analysis		Multivariate Analysis	
		HR (95% CI)	*P* value	HR (95% CI)	*P* value
Stage II/III	SAMHD1_high vs. SAMHD1_low	1.82 (0.94-3.56)	0.078	1.55 (0.77-3.09)	0.217
	Age^a^	1.04 (1.01-1.07)	0.002	1.05 (1.02-1.07)	0.001
	Male vs. Female	0.79 (0.46-1.37)	0.409	0.75 (0.43-1.32)	0.327
	Stage III vs. Stage II	2.20 (1.25-3.85)	0.006	2.58 (1.47-4.55)	0.001
Stage II	SAMHD1_high vs. SAMHD1_low	2.89 (1.17-7.18)	0.022	2.99 (1.17-7.65)	0.023
	Age^a^	1.12 (1.05-1.18)	<0.001	1.12 (1.05-1.20)	<0.001
	Male vs. Female	1.09 (0.46-2.58)	0.849	1.20 (0.49-2.94)	0.686
Stage III	SAMHD1_high vs. SAMHD1_low	1.27 (0.44-3.67)	0.653	0.92 (0.30-2.80)	0.888
	Age^a^	1.03 (1.00-1.06)	0.086	1.03 (1.00-1.06)	0.055
	Male vs. Female	0.70 (0.34-1.45)	0.334	0.59 (0.28-1.27)	0.180

^a^continuous variable.

CI, confidence interval; HR, hazard ratio; SAMHD1, sterile alpha motif and histidine-aspartate domain-containing protein 1.

### SAMHD1 expression and OS in the validation dataset

To evaluate the robustness of our findings, we performed an analysis in the validation dataset including 56 SAMHD1-high patients (12.04%) and 409 SAMHD1-low patients (87.96%). The baseline characteristics and known molecules of stage II and III in the validation dataset were described in **Table S7**. As shown in **Figure 2B**, we observed that the high expression of SAMHD1 (n = 29) was associated with a lower 5-year OS rate than a low expression of SAMHD1 (n = 232) among stage II patients (HR = 2.25; 95% CI, [1.17–4.34]; and *P* = 0.013), but not in stage III patients (n = 204; HR = 0.64; 95% CI, [0.27–1.50]; and *P* = 0.299) (**Table 1**). After adjusting for sex, age, and adjuvant chemotherapy, multivariate analysis also confirmed that high SAMHD1 expression status was associated with shorter OS in stage II patients (HR = 2.81; 95% CI, [1.43–5.50]; and *P* = 0.003) (**Table 2**).

**Table 2:** Univariate and multivariable Cox analyses for overall survival among patients in the validation data set.

Subgroup	Variable	Univariate Analysis		Multivariate Analysis	
		HR (95% CI)	*P* value	HR (95% CI)	*P* value
Stage II/III	SAMHD1_high vs. SAMHD1_low	1.27 (0.76-2.13)	0.359	1.14 (0.66-1.95)	0.634
	Age^a^	1.03 (1.02-1.05)	<0.001	1.03 (1.01-1.05)	0.001
	Male vs. Female	1.28 (0.88-1.86)	0.195	1.47 (1.01-2.16)	0.046
	Stage III vs. Stage II	1.30 (0.90-1.88)	0.156	1.70 (1.08-2.67)	0.021
	Adjuvant Chemotherapy^b^	0.71 (0.49-1.03)	0.073	0.64 (0.40-1.04)	0.071
Stage II	SAMHD1_high vs. SAMHD1_low	2.25 (1.17-4.34)	0.015	2.81 (1.43-5.50)	0.003
	Age^a^	1.03 (1.01-1.05)	0.016	1.04 (1.01-1.06)	0.006
	Male vs. Female	1.20 (0.71-2.04)	0.496	1.32 (0.78-2.25)	0.301
	Adjuvant Chemotherapy^b^	0.76 (0.40-1.47)	0.414	1.07 (0.53-2.16)	0.850
Stage III	SAMHD1_high vs. SAMHD1_low	0.64 (0.27-1.50)	0.304	0.43 (0.18-1.02)	0.056
	Age^a^	1.03 (1.01-1.06)	0.002	1.03 (1.01-1.05)	0.017
	Male vs. Female	1.43 (0.84-2.44)	0.185	1.60 (0.93-2.75)	0.088
	Adjuvant Chemotherapy^b^	0.40 (0.24-0.68)	0.001	0.40 (0.22-0.71)	0.002

^a^continuous variable. ^b^yes vs. no.

CI, confidence interval; HR, hazard ratio; SAMHD1, sterile alpha motif and histidine-aspartate domain-containing protein 1.

The SAMHD1 expression groups had similar hazard ratios among stage II patients compared with the classical risk parameter such as age and T stage in the multivariate Cox regression analyses (**Figure S11**), which is based on analyses about the relative importance of each risk parameter for OS using the x² proportion test in stage II patients.

### SAMHD1 expression and benefit from adjuvant chemotherapy

**Figure 3:**
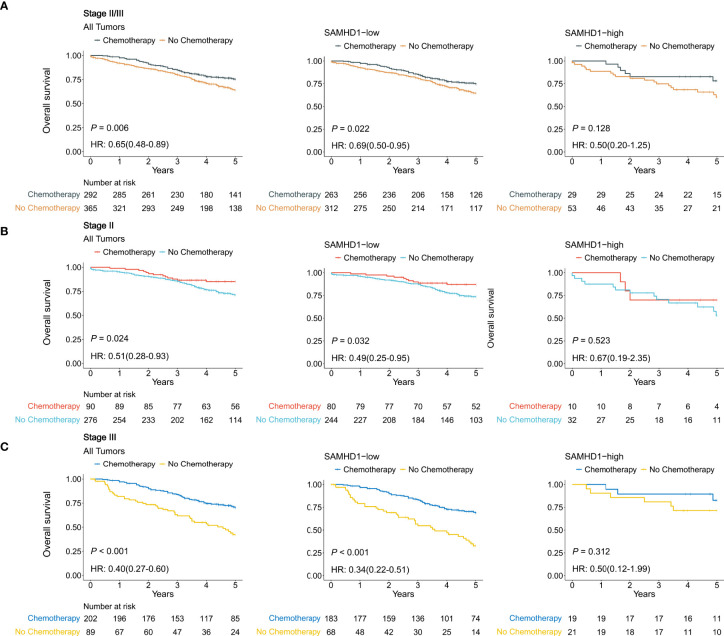
Relationship between SAMHD1 expression and the benefit from adjuvant chemotherapy using Gene Expression Omnibus data. **(A)** Patients with stage II and stage III disease. **(B)** Patients with stage II disease. **(C)** Patients with stage III disease.

**Figure 3** shows the relationship between the expression levels of SAMHD1 and benefit from adjuvant chemotherapy in 657 patients with stage II or III colorectal cancer. In the SAMHD1-low patient population, treatment with adjuvant chemotherapy was associated with higher OS in the stage II subgroup (88% with chemotherapy vs. 77% with no chemotherapy; HR = 0.49; 95% CI, [0.25–0.95], and *P* = 0.032) and in the stage III subgroup (73% with chemotherapy vs. 44% with no chemotherapy; HR = 0.34; 95% CI, [0.22–0.51], and *P* < 0.001) (**Figure 3**). In the SAMHD1-high patient population, treatment with adjuvant chemotherapy was not associated with higher OS in either the stage II subgroup (chemotherapy vs. no chemotherapy; HR = 0.67; 95% CI, [0.19–2.35], and *P* = 0.523) or the stage III subgroup (chemotherapy vs. no chemotherapy, HR = 0.50; 95% CI, [0.12–1.99], and *P* = 0.312) (**Figure 3**).

## Discussion

Using proteomics analysis, SAMHD1 was identified as a potential biomarker displaying a significant prognostic value. It was differentially expressed in the paired colorectal cancer groups with and without recurrence as selected by a nested case-control design from a large retrospective cohort. Using public colorectal cancer datasets for biomarker discovery, we illustrated that SAMHD1 had prognostic and predictive powers that could be helpful for patients with stage II colorectal cancer and had a predictive power in those with stage III colorectal cancer. Validation was performed using tissue microarrays on different cohorts of patients. Hence, SAMHD1 could complement MSI/MMR status as a molecular marker involved in the high-risk definition for patients with stage II colorectal cancer and help in making clinical decisions for adjuvant chemotherapy for patients with stages II and III colorectal cancer.

This study showed that the high expression of SAMHD1 in stage II colorectal cancer tissues was correlated with poor prognosis. We speculated that a higher rate of mutations may occur in patients with high expression of SAMHD1 resulting in disease progression because mutations in SAMHD1 that alter its dNTPase activity are associated with colon cancer (40). Moreover, a previous study reported that SAMHD1 upregulation was found in the colorectal cancer tissue of the patients with advanced colorectal cancer compared to their normal counterparts (41). Additionally, the role of SAMHD1 in numerous types of cancer, such as chronic lymphocytic leukemia, lung cancer, and colorectal cancer, has been extensively studied (42). Moreover, the high expression level of SAMHD1 had an independent significant association with unfavorable OS in some types of cancer (37, 43, 44). Hence, the expression level of SAMHD1 could be a prognostic biomarker for stage II colorectal cancer.

This study is the first to demonstrate that SAMHD1 is a predictive biomarker for adjuvant chemotherapy in patients with stage II and III colorectal cancer. Several studies have reported that high expression of SAMHD1 negatively impacts the efficacy of nucleoside-based chemotherapies in different cohorts of patients with leukemia (36, 37, 43, 45–47). The negative role of SAMHD1 in the sensitivity to chemotherapy can be attributed to various reasons. SAMHD1 is a dNTPase that hydrolyzes dNTPs into deoxyribonucleosides (dNs) and triphosphates (48). It has been identified as a restriction factor that blocks infection by a broad range of retroviruses, including HIV-1, in noncycling myeloid-lineage cells and quiescent CD4+ T lymphocytes (49–54). Owing to its dNTPase activity, SAMHD1 can degrade the analog cytarabine triphosphate and reduce its concentrations in cells, such as the patient-derived acute myeloid leukemia blasts, thereby posing a significant barrier to the effective analog cytarabine-based treatment (45). However, SAMHD1 can hydrolyze several active triphosphate (TP) nucleoside analogs used for anti-cancer therapies (47). Therefore, evaluation of the expression levels of SAMHD1 in patients with stages II and III colorectal cancer before adjuvant chemotherapy is warranted.

SAMHD1 could complement MSI/MMR status as a promising molecular marker, leading to more accurate treatment decisions in patients with stage II colorectal cancer. The MSI/MMR status of the tumor is the only molecular marker involved in adjuvant chemotherapy decisions for stage II colorectal cancer (11). However, the MSI/MMR rate is 10%–15%, while 20% of patients with stage II colorectal cancer experience relapse after surgery (3). This leads to the emerging need to identify novel biomarkers for the effective treatment of colorectal cancer. Our results show that SAMHD1 expression only partially overlaps with tumors defined by the MSI/MMR status. In this study, high expression of SAMHD1 was approximately 12% and conferred poor prognosis and less benefits from adjuvant chemotherapy for stage II disease in both the discovery and validation datasets. We will further assess the prognostic and predictive value of SAMHD1 using immunohistochemistry in a prospective multicenter cohort before clinical practice.

The major strength of our study is its nested case-control design combined with proteomics. The nested case-control design is an efficient method to identify novel prognostic biomarkers using the available, large sets of clinical data storing biological samples and taking both feasibility and economic factors into account (55). We identified 21 proteins associated with the prognosis of patients with colorectal cancer by using quantitative proteomics in a nested case-control cohort within a large cohort of patients with colorectal cancer. Among these 21 proteins, five proteins showed a promising role as potential biomarkers for the identification of high-risk populations and chemo-sensitive patients with stage II colorectal cancer. Therefore, further studies are required to validate these results.

Each omics discipline has its own advantages and disadvantages, and can give information about many aspects of disease from transcriptomics signatures to proteomic profiles. By comparison, colorectal cancer-related protein-coding genes have little overlap with known cancer genes, this is one of the advantages of proteomics over other omics (29, 56). It is logical therefore to examine this extensive information in parallel with the aim of revealing those attributes that can be considered robust and sensitive enough to work as a biomarker of patient risk (57).

While the results are promising, this study has several limitations. Firstly, this study was lack of immunohistochemical validation of SAMHD1 due to the retrospective design, we could not obtain effective FFPE specimen from many patients because of the long storage time. We will further validate these results using the prospective, multicenter clinical trials. Secondly, since there were few stage III patients in our cohort, we do get lose the predictivity in stage III patients, the specific reasons are not clear, and further research is needed in the future. Thirdly, we did not perform more detailed analysis about adjuvant chemotherapy regimens due to the lack of specific treatment information in public datasets, so SAMHD1 should be a predictive parameter for a group of drugs, and much more clinical data should be available until SAMHD1 could be an add-on to clinical practice.

In conclusion, our research showed that SAMHD1 can effectively stratify patients with stage II colorectal cancer into subgroups with good and poor prognosis, thereby complementing the prognostic value of the MSI/MMR status that is used to evaluate the prognosis of these patients. Moreover, our results showed that the expression levels of SAMHD1 can identify stages II and III patients who could benefit from adjuvant chemotherapy. Thus, SAMHD1 may potentially be used as an easy and useful tool in clinical practice to develop more accurate treatment decisions for patients with stages II and III colorectal cancer.

## Data availability statement

The datasets presented in this study can be found in online repositories. The names of the repository/repositories and accession number(s) can be found in the article/**Supplementary material**.

## Ethics statement

The studies involving human participants were reviewed and approved by Yunnan Cancer Hospital Ethics Committee (No. KY2019141). The patients/participants provided their written informed consent to participate in this study. Written informed consent was obtained from the individual(s) for the publication of any potentially identifiable images or data included in this article.

## Author contributions

DY, SZ, and SY helped design the statistical approach, performed all the analyses, interpreted the results, and drafted the manuscript. YD, XC, LW, and WW helped with the collection of patient-related data. CL helped with the statistical methodology. TZ, ZL, and YH conceptualized and led the study, helped design the study and interpret the results, and revised and submitted the manuscript. All authors approved the final manuscript.

## Funding

This study was supported by the National Natural Scientific Foundation of China (82001986, 82073569, 81973147), National Science Fund for Distinguished Young Scholars (81925023), the Outstanding Youth Science Foundation of Yunnan Basic Research Project (202101AW070001, 202001AW070021), the Key Science Foundation of Yunnan Basic Research (202101AS070040), Yunnan Digitalization, Development and Application of Biotic Resource (202002AA100007), the Innovation Team of Kunming Medical University (CXTD202110).

## Acknowledgments

The authors thank the patients for making this study possible.

## Conflict of interest

The authors declare that the research was conducted in the absence of any commercial or financial relationships that could be construed as a potential conflict of interest.

## Publisher’s note

All claims expressed in this article are solely those of the authors and do not necessarily represent those of their affiliated organizations, or those of the publisher, the editors and the reviewers. Any product that may be evaluated in this article, or claim that may be made by its manufacturer, is not guaranteed or endorsed by the publisher.
