# Supplementary material for: SAMHD1 as a prognostic and predictive biomarker in stage II colorectal cancer: A multicenter cohort study
Source: Front Oncol. 2022 Aug 1;12:939982. doi: 10.3389/fonc.2022.939982 (PMC9376296; doi:10.3389/fonc.2022.939982)
Supplement: Supplementary file 1 [file DataSheet_1.docx]

Supplementary Material

# Supplementary Figures and Tables

## Supplementary Figures

**Figure S1.** Patients’ composition of the “Discovery Dataset” - The Cancer Genome Atlas.

**Figure S2.** Patients’ composition of the “Validation Dataset” - GSE40967.

**Figure S3.** Patients’ composition of the “Expansion Dataset #1” - GSE29623.

**Figure S4.** Patients’ composition of the “Expansion Dataset #2” - GSE103479.

**Figure S5**. Survival curves showing overall survival and recurrence-free survival among 11 paired patients after propensity score matching. (A) Overall survival. (B) Recurrence-free survival.

**Figure S6**. Investigation of the significant proteins. (A) Volcano plot showing the significant proteins (red nodes) and non-significant proteins (blue nodes). (B) Venn diagram showing the intersection of cox regression and t-test for differentially expressed proteins.

**Figure S7**. Violin Diagram Showing the Distribution of SAMHD1 Protein Expression. Left: The distribution of SAMHD1 protein expression between the metastatic and non-metastatic groups. Right: Tumor and adjacent tissues.

**Figure S8**. Scatterplots and Pearson’s correlation analysis showing the relationships between SAMHD1 gene expression and expression of genes associated with microsatellite instability (MLH1, MSH2, MSH6, PMS2), using The Cancer Genome Atlas dataset. The correlation coefficients and *P*-values are shown in each plot.

**Figure S9** Beeswarms and Boxplots Showing the distribution of SAMHD1 Gene Expression in GSE40967. The distribution of SAMHD1 gene expression between (A) BRAF wild type and BRAF mutant group. (B) KRAS wild type and KRAS mutant group. (C) Distal and proximal tissues. (D) pMMR status and dMMR status. WT, wild type; M, mutant.

**Figure S10**. X-tile plots of SAMHD1 gene expression in stage II patients from The Cancer Genome Atlas dataset.


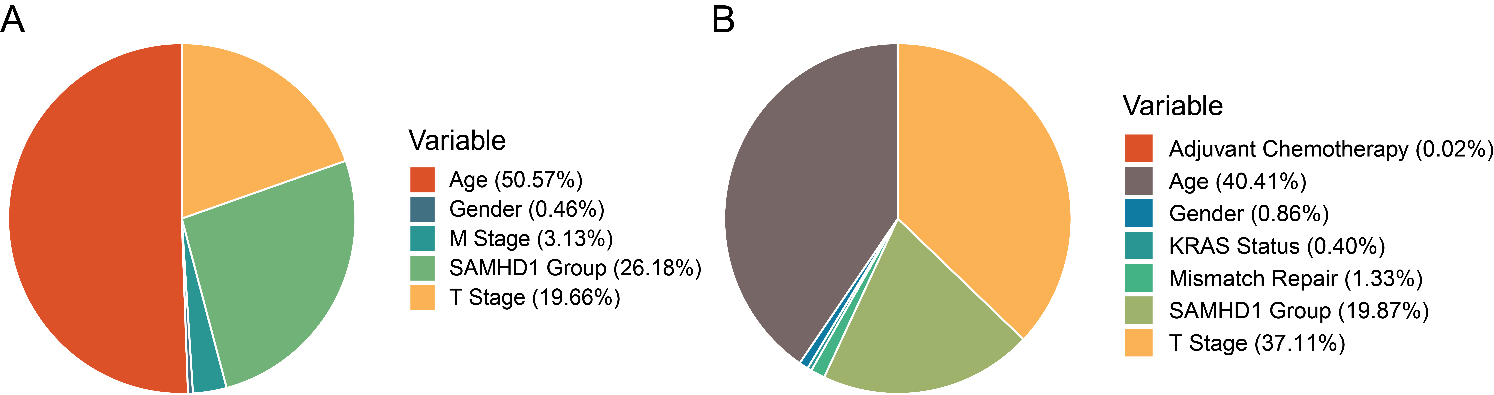


**Figure S11**. Relative importance of each risk parameter for OS using the 𝜒² proportion test in TCGA (A) and GSE40967 (B).

## Supplementary Tables

**Table S1.** Characteristics of patients at baseline and follow-up in the discovery and validation data sets

**Table S2.** Characteristics of 11 paired patients at baseline and follow-up

**Table S3.** The list of the t-test analysis results of 2,760 proteins

**Table S4.** The list of the univariate COX analysis results of 2,760 proteins

**Table S5.** The list of differentially expressed proteins

**Table S6.** Characteristics of patients with stage II and III at baseline and follow-up in the TCGA discovery dataset.

**Table S7.** Characteristics of patients with stage II and III at baseline and follow-up in the GEO validation dataset.

## Supplementary Material A
