# Supplementary material for: SAMHD1 as a prognostic and predictive biomarker in stage II colorectal cancer: A multicenter cohort study
Source: Front Oncol. 2022 Aug 1;12:939982. doi: 10.3389/fonc.2022.939982 (PMC9376296; doi:10.3389/fonc.2022.939982)
Supplement: Supplementary file 2 [file DataSheet_2.docx]

**Supplementary Material A**

**Protein extraction**

For each sample, protein was extracted using FFPE Total Protein Extraction Kit (Sangon Biotech, NO. C500058, Shanghai, China) according to the manual. The extracted protein was quantified with a BCA Protein Assay Kit (Bio-Rad, USA).

**Protein Digestion and** **TMT Labeling**

Protein digestion was performed according to the FASP procedure described by Wiśniewski et al.^[1]^ and the resulting peptide mixture was labeled using the 6-plex TMT reagent according to the manufacturer’s instructions (Thermo Fisher). Briefly, 200 μg of proteins for each sample were incorporated into 30 μl STD buffer (4% SDS, 100 mM DTT, 150 mM Tris-HCl pH 8.0). The detergent, DTT and other low-molecular-weight components were removed using UA buffer (8 M Urea, 150 mM Tris-HCl pH 8.0) by repeated ultrafiltration (Microcon units, 30 kD). Then 100 μl 0.05 M iodoacetamide in UA buffer was added to block reduced cysteine residues and the samples were incubated for 20 min in darkness. The filters were washed with 100 μl UA buffer three times and then 100 μl DS buffer (50 mM triethylammoniumbicarbonate at pH 8.5) twice. Finally, the protein suspensions were digested with 2 μg trypsin (Promega) in 40 μl DS buffer overnight at 37 °C, and the resulting peptides were collected as a filtrate. The peptide content was estimated by UV light spectral density at 280 nm using an extinctions coefficient of 1.1 of 0.1% (g/l) solution that was calculated on the basis of the frequency of tryptophan and tyrosine in vertebrate proteins.

For labeling, each TMT reagent was dissolved in 70 μl of ethanol and added to the respective peptide mixture. The samples were labeled as is shown in Table 1, and were multiplexed and vacuum dried.

Table 1．The label of the sample.

| No. | | | | | |
| --- | --- | --- | --- | --- | --- |
| 1 | | 2 | | 3 | |
| Tissue name | TMT label | Tissue name | TMT label | Tissue name | TMT label |
| 0997-cancer | 126 | 2377-cancer | 126 | 1400-cancer | 126 |
| 0997-normal | 127N | 2377-normal | 127N | 1400-normal | 127N |
| 1446-cancer | 127C | 2512-cancer | 127C | 2681-cancer | 127C |
| 1446-normal | 128N | 2512-normal | 128N | 2681-normal | 128N |
| 1090-cancer | 128C | 1110-cancer | 128C | 1745-cancer | 128C |
| 1090-normal | 129N | 1110-normal | 129N | 1745-normal | 129N |
| 1895-cancer | 129C | 1254-cancer | 129C | 2007-cancer | 129C |
| 1896-normal | 130N | 1254-normal | 130N | 2007-normal | 130N |
| 0543-cancer | 130C | 1129-cancer | 130C | 1382-cancer | 130C |
| 0543-normal | 131N | 1129-normal | 131N | 1382-normal | 131N |
| 1619-cancer | 131C | 1421-cancer | 131C | 2798-cancer | 131C |
| 1619-normal | 132N | 1421-normal | 132N | 2798-normal | 132N |
| 0255-cancer | 132C | 1292-cancer | 132C | 1643-cancer | 132C |
| 0255-normal | 133N | 1292-normal | 133N | 1643-normal | 133N |
| 1060-cancer | 133C | 1641-cancer | 133C | 2189-cancer | 133C |
| 1060-normal | 134N | 1641-normal | 134N | 2189-normal | 134N |

**Peptide Fractionation with Strong Cation Exchange (SCX) Chromatography**

TMT labeled peptides were fractionated by SCX chromatography using the AKTA Purifier system (GE Healthcare). The dried peptide mixture was reconstituted and acidified with 2 ml buffer A (10 mM KH_2_PO_4_ in 25% of ACN, pH 2.7) and loaded onto a PolySULFOETHYL 4.6 x 100 mm column (5 µm, 200 Å, PolyLC Inc, Maryland, U.S.A.). The peptides were eluted at a flow rate of 1 ml/min with a gradient of 0%–10% buffer B (500 mM KCl, 10 mM KH_2_PO_4_ in 25% of ACN, pH 2.7) for 2 min, 10–20% buffer B for 25 min, 20%–45% buffer B for 5 min, and 50%–100% buffer B for 5 min. The elution was monitored by absorbance at 214 nm, and fractions were collected every 1 min. The collected fractions (about 30 fractions) were finally combined into 10 pools and desalted on C18 Cartridges (Empore™ SPE Cartridges C18 (standard density), bed I.D. 7 mm, volume 3 ml, Sigma). Each fraction was concentrated by vacuum centrifugation and reconstituted in 40 µl of 0.1% (v/v) trifluoroacetic acid. All samples were stored at -80°C until LC-MS/MS analysis.

**Liquid Chromatography (LC) - Electrospray Ionization (ESI) Tandem MS (MS/MS) Analysis by Q Exactive.**

Experiments were performed on a Q Exactive mass spectrometer that was coupled to Easy nLC (Proxeon Biosystems, now Thermo Fisher Scientific). 10 μl of each fraction was injected for nanoLC-MS/MS analysis. The peptide mixture (5 μg) was loaded onto a the C18-reversed phase column (15 cm long, 75 μm inner diameter) packed in-house with RP-C18 5μm resin in buffer A (0.1% Formic acid) and separated with a linear gradient of buffer B (80% acetonitrile and 0.1% Formic acid) at a flow rate of 250 nl/min controlled by IntelliFlow technology over 140 min. MS data was acquired using a data-dependent top10 method dynamically choosing the most abundant precursor ions from the survey scan (300–1800 m/z) for HCD fragmentation. Determination of the target value is based on predictive Automatic Gain Control (pAGC). Dynamic exclusion duration was 60 s. Survey scans were acquired at a resolution of 70,000 at m/z 200 and resolution for HCD spectra was set to 17,500 at m/z 200. Normalized collision energy was 30 eV and the underfill ratio, which specifies the minimum percentage of the target value likely to be reached at maximum fill time, was defined as 0.1%. The instrument was run with peptide recognition mode enabled.

**Sequence Database Searching and Data Analysis**

MS/MS spectra were searched using MASCOT engine (Matrix Science, London, UK; version 2.2) embedded into Proteome Discoverer 1.3 (Thermo Electron, San Jose, CA.) against UniProt_Homo_sapiens_192367_20200810 (192367 sequences, download at 2020/08/10) and the decoy database. For protein identification, the following options were used. Peptide mass tolerance = 20 ppm, MS/MS tolerance = 0.1 Da, Enzyme = Trypsin, Missed cleavage = 2, Fixed modification: Carbamidomethyl (C), TMT 16plex(K), TMT 16plex(N-term), Variable modification：Oxidation(M), FDR ≤ 0.01.

**REFERENCE**

1. Wiśniewski JR, Zougman A, Nagaraj N, Mann M: **Universal sample preparation method for proteome analysis**. *Nat Methods* 2009, **6**(5):359-362.
