# Supplementary material for: SAMHD1 as a prognostic and predictive biomarker in stage II colorectal cancer: A multicenter cohort study
Source: Front Oncol. 2022 Aug 1;12:939982. doi: 10.3389/fonc.2022.939982 (PMC9376296; doi:10.3389/fonc.2022.939982)
Supplement: Supplementary file 3 [file Table_1.docx]

| **Table S1.** Characteristics of patients at baseline and follow-up in the discovery and validation data sets. | | | | |
| --- | --- | --- | --- | --- |
| **Variable** | **TCGA (n = 335)** | **GSE40967 (n = 465)** | **GSE29623 (n = 40)** | **GSE103479 (n = 152)** |
| Baseline |  |  |  |  |
| Age, year ^a^ | 68.0 [58.0, 76.0] | 69.0 [59.0, 77.0] | N/A^a^ | 70.7 [62.1, 78.2] |
| Male, n (%) | 181 (54.0) | 260 (55.9) | 22 (55.0) | 84 (55.3) |
| Stage, n (%) |  |  |  |  |
| II | 193 (57.6) | 261 (56.1) | 22 (55.0) | 83 (54.6) |
| III | 142 (42.4) | 204 (43.9) | 18 (45.0) | 69 (45.4) |
| T Stage, n (%) |  |  |  |  |
| T1 | 0 (0.0) | 2 (0.4) | 0 (0.0) | 1 (0.7) |
| T2 | 9 (2.7) | 19 (4.1) | 1 (2.5) | 6 (3.9) |
| T3 | 295 (88.1) | 332 (71.4) | 36 (90.0) | 110 (72.4) |
| T4 | 31 (9.3) | 92 (19.8) | 3 (7.5) | 35 (23.0) |
| N/A ^b^ | 0 (0.0) | 20 (4.3) | 0 (0.0) | 0 (0.0) |
| N Stage, n (%) |  |  |  |  |
| N0 | 193 (57.6) | 250 (53.8) | 21 (52.5) | 83 (54.6) |
| N1 | 91 (27.2) | 110 (23.7) | 15 (37.5) | 48 (31.6) |
| N2 | 51 (15.2) | 75 (16.1) | 3 (7.5) | 21 (13.8) |
| N3 | 0 (0.0) | 10 (2.2) | 0 (0.0) | 0 (0.0) |
| NX | 0 (0.0) | 0 (0.0) | 1 (2.5) | 0 (0.0) |
| N/A ^b^ | 0 (0.0) | 20 (4.3) | 0 (0.0) | 0 (0.0) |
| M Stage, n (%) |  |  |  |  |
| M0 | 297 (88.7) | 443 (95.3) | 39 (97.5) | 86 (56.6) |
| M1 | 0 (0.0) | 1 (0.2) | 0 (0.0) | 0 (0.0) |
| MX | 34 (10.1) | 1 (0.2) | 1 (2.5) | 66 (43.4) |
| N/A ^b^ | 4 (1.2) | 20 (4.3) | 0 (0.0) | 0 (0.0) |
| Adjuvant Chemotherapy, n (%) | N/A^a^ | 203 (43.7) | 23 (57.5) | 66 (43.4) |
| Follow-Up |  |  |  |  |
| Overall Survival, n (%) | 277 (82.7) | 320 (68.8) | 28 (70.0) | 93 (61.2) |
| Overall Survival Time, year ^a^ | 1.9 [1.1, 3.1] | 4.6 [2.7, 6.8] | 4.5 [3.1, 5.5] | 4.3 [3.2, 7.0] |
| Note: ^a^, data are median [IQR], or n (%); ^b^, missing value.  GEO, Gene Expression Omnibus; TCGA, Cancer Genome Atlas. | | | | |
