# Supplementary material for: SAMHD1 as a prognostic and predictive biomarker in stage II colorectal cancer: A multicenter cohort study
Source: Front Oncol. 2022 Aug 1;12:939982. doi: 10.3389/fonc.2022.939982 (PMC9376296; doi:10.3389/fonc.2022.939982)
Supplement: Supplementary file 4 [file Table_2.docx]

**Table S2**. Characteristics of 11 paired patients at baseline and follow-up.

| **Variable** | **Non-Metastasis (n = 11)** | **Metastasis (n = 11)** | ***P*-value** |
| --- | --- | --- | --- |
| **Baseline** |  |  |  |
| Age, year | 61.0 [53.5, 65.5] | 56.0 [50.0, 67.0] | 0.866 |
| BMI, kg/m^2^ | 21.2 [20.9, 23.4] | 21.3 [20.2, 23.4] | 0.929 |
| Male, n (%) | 5 (45.5) | 5 (45.5) | 1.000 |
| Preoperative CEA, μg/L | 2.3 [1.1, 6.7] | 5.4 [3.6, 27.8] | 0.108 |
| Preoperative CA125, U/ml | 12.7 [6.9, 13.6] | 18.4 [15.4, 22.7] | 0.006 |
| Preoperative CA199, U/mL | 11.7 [8.6, 26.5] | 17.1 [10.9, 21.4] | 0.718 |
| Stage, n (%) |  |  |  |
| II | 8 (72.7) | 8 (72.7) | 1.000 |
| III | 3 (27.3) | 3 (27.3) |  |
| T Stage, n (%) |  |  |  |
| T3 | 11 (100.0) | 11 (100.0) | NA |
| N Stage, n (%) |  |  |  |
| N0 | 8 (72.7) | 8 (72.7) | 1.000 |
| N1 | 3 (27.3) | 3 (27.3) |  |
| Follow-Up |  |  |  |
| Overall Survival, n (%) | 10 (90.9) | 6 (54.5) | 0.151 |
| Overall Survival Time, month | 51.6 [41.9, 57.9] | 31.8 [19.6, 44.0] | 0.002 |
| Recurrence-free Survival, n (%) | 11 (100.0) | 6 (54.5) | 0.042 |
| Recurrence-free Survival Time, month | 51.6 [41.9, 57.9] | 21.4 [14.9, 32.0] | <0.001 |

Note：Data are median [IQR], or n (%)
