# Supplementary material for: SAMHD1 as a prognostic and predictive biomarker in stage II colorectal cancer: A multicenter cohort study
Source: Front Oncol. 2022 Aug 1;12:939982. doi: 10.3389/fonc.2022.939982 (PMC9376296; doi:10.3389/fonc.2022.939982)
Supplement: Supplementary file 8 [file Table_6.docx]

**Table S6.** Characteristics of patients with stage II and III at baseline and follow-up in the TCGA discovery data sets.

| **Variable** | **Stage II (n = 193)** | **Stage III (n = 142)** | ***P* value** |
| --- | --- | --- | --- |
| Age, year ^a^ | 68.0 [60.0, 77.0] | 65.0 [55.0, 73.8] | 0.013 |
| Male, n (%) | 109 (56.5) | 72 (50.7) | 0.349 |
| T Stage, n (%) |  |  |  |
| T2 | 0 (0.0) | 9 (6.3) | <0.001 |
| T3 | 180 (93.3) | 115 (81.0) |  |
| T4 | 13 (6.7) | 18 (12.7) |  |
| N Stage, n (%) |  |  |  |
| N0 | 193 (100.0) | 0 (0.0) | <0.001 |
| N1 | 0 (0.0) | 91 (64.1) |  |
| N2 | 0 (0.0) | 51 (35.9) |  |
| M Stage, n (%) |  |  |  |
| M0 | 179 (92.7) | 118 (83.1) | 0.019 |
| MX | 12 (6.2) | 22 (15.5) |  |
| N/A^b^ | 2 (1.0) | 2 (1.4) |  |
| Follow-Up |  |  |  |
| Overall Survival, n (%) | 28 (14.5) | 30 (21.1) | 0.151 |
| Overall Survival Time, year ^a^ | 2.0 [1.1, 3.3] | 1.8 [1.2, 2.7] | 0.154 |
| SAMHD1-high, n (%) | 27 (14.0) | 15 (10.6) | 0.442 |

Note: a, data are median [IQR], or n (%); b, missing value.
