# Supplementary material for: SAMHD1 as a prognostic and predictive biomarker in stage II colorectal cancer: A multicenter cohort study
Source: Front Oncol. 2022 Aug 1;12:939982. doi: 10.3389/fonc.2022.939982 (PMC9376296; doi:10.3389/fonc.2022.939982)
Supplement: Supplementary file 9 [file Table_7.docx]

**Table S7.** Characteristics of patients with stage II and III at baseline and follow-up in the GEO validation data sets.

| **Variable** | **Stage II (n = 261)** | **Stage III (n = 204)** | ***P* value** |
| --- | --- | --- | --- |
| Age, year ^a^ | 70.0 [61.0, 77.0] | 68.0 [59.0, 76.1] | 0.603 |
| Male, n (%) | 154 (59.0) | 106 (52.0) | 0.154 |
| T Stage, n (%) |  |  | < 0.001 |
| T1 | 0 (0.0) | 2 (1.0) |  |
| T2 | 4 (1.5) | 15 (7.4) |  |
| T3 | 195 (74.7) | 137 (67.2) |  |
| T4 | 51 (19.5) | 41 (20.1) |  |
| N/A ^b^ | 11 (4.2) | 9 (4.4) |  |
| N Stage, n (%) |  |  | < 0.001 |
| N0 | 250 (95.8) | 0 (0.0) |  |
| N1 | 0 (0.0) | 110 (53.9) |  |
| N2 | 0 (0.0) | 75 (36.8) |  |
| N3 | 0 (0.0) | 10 (4.9) |  |
| N/A ^b^ | 11 (4.2) | 9 (4.4) |  |
| M Stage, n (%) |  |  | 0.557 |
| M0 | 249 (95.4) | 194 (95.1) |  |
| M1 | 1 (0.4) | 0 (0.0) |  |
| MX | 0 (0.0) | 1 (0.5) |  |
| N/A ^b^ | 11 (4.2) | 9 (4.4) |  |
| Adjuvant Chemotherapy, n (%) | 56 (21.5) | 147 (72.1) | < 0.001 |
| Follow-Up |  |  |  |
| Overall Survival, n (%) | 78 (29.9) | 67 (32.8) | 0.560 |
| Overall Survival Time, year ^a^ | 4.8 [2.8, 7.1] | 4.4 [2.5, 6.5] | 0.066 |
| SAMHD1-high, n (%) | 29 (11.1) | 29 (14.2) | 0.388 |
| BRAF Status, n (%) |  |  | < 0.001 |
| Wild type | 224 (85.8) | 146 (71.6) |  |
| Mutated | 20 (7.7) | 21 (10.3) |  |
| N/A ^b^ | 17 (6.5) | 37 (18.1) |  |
| KRAS Status, n (%) |  |  | 0.053 |
| Wild type | 166 (63.6) | 110 (53.9) |  |
| Mutated | 87 (33.3) | 81 (39.7) |  |
| N/A ^b^ | 8 (3.1) | 13 (6.4) |  |
| MMR Status ^c^, n (%) |  |  | < 0.001 |
| Deficient MMR ^c^ | 36 (13.8) | 24 (11.8) |  |
| Proficient MMR ^c^ | 184 (70.5) | 177 (86.8) |  |
| N/A ^b^ | 41 (15.7) | 3 (1.5) |  |

Note: a, data are median [IQR], or n (%); b, missing value; c, mismatch repair.
